# Regulation of Cancer Stem Cells in the Tumor Microenvironment

**DOI:** 10.47248/chp2502040016

**Published:** 2025-09-26

**Authors:** Aidan Li, Na Li, Yajing Yang, Linzhou Wang, Jessica Miao, Qi-En Wang

**Affiliations:** 1. Department of Radiation Oncology, College of Medicine, The Ohio State University, Columbus, OH 43210, USA;; 2. Comprehensive Cancer Center, The Ohio State University, Columbus, OH 43210, USA

**Keywords:** Cancer stem cells, tumor microenvironment, cellular plasticity, CSC niche

## Abstract

Cancer stem cells (CSCs) represent a subpopulation of cancer cells characterized by their capacity for self-renewal, differentiation, and tumorigenicity. CSCs exist along a spectrum of stemness regulated by both intrinsic factors and extrinsic signals from the tumor microenvironment (TME). The TME is composed of diverse cell types such as stromal and immune cells, and also physical factors such as the extracellular matrix and hypoxia. Environmental signals originating from the TME can induce non-CSCs to acquire stem-like traits, while CSCs in turn modulate the TME by recruiting and reprogramming immune and stromal cells. Analogous to normal stem cell niches, CSCs reside in or construct supportive niches that promote stemness, metastasis, immune evasion, and therapy resistance. This reciprocal interaction between CSCs and the TME underscores the complexity of cancer stemness and presents challenges and opportunities for therapeutics.

## 1. Introduction

Cancer stem cells (CSCs) are a subset of cells within the heterogeneous population of tumoral cells that are distinguished by their ability to self-renew and differentiate into other cancer cell subpopulations [1]. The concept of CSCs originally arose from the identification of cancer cell subpopulations exhibiting highly tumorigenic properties [2,3]. Early models of CSCs leaned toward a discrete, binary classification: cancer cells either were non-CSCs or CSCs, with CSCs defined by the ability to self-renew, differentiate, and divide either symmetrically or asymmetrically [4]. Further work characterizing the other properties of CSCs, such as drug resistance and metastatic potential [5,6], combined with identification of the pathways contributing to CSC maintenance, such as Wnt, Notch, and Hedgehog signaling [7], has led to the refinement and development of new frameworks for understanding CSCs. Within tumors, cancer cells are now widely understood to lie along a dynamic spectrum ranging from highly differentiated cells to those exhibiting varying levels of stemness [8]. Like normal human stem cells, CSC stemness is controlled by a highly complex network of environmental signals, where non-CSCs can be directed to take on a more stem-like phenotype and vice versa [9]. Accordingly, methods for evaluating cancer cell stemness involve *in vitro* assessment of sphere/colony formation, stemness markers such as SOX2, Nanog, and Oct4, and *in vivo* tumorigenesis [10]. Cell sorting techniques to evaluate aldehyde dehydrogenase (ALDH) activity and cell surface markers such as CD44 and CD133 are also used to determine and isolate CSCs, but specific CSC marker sets vary by cancer type and may also be expressed by non-cancer cells [11].

**Figure 1:**
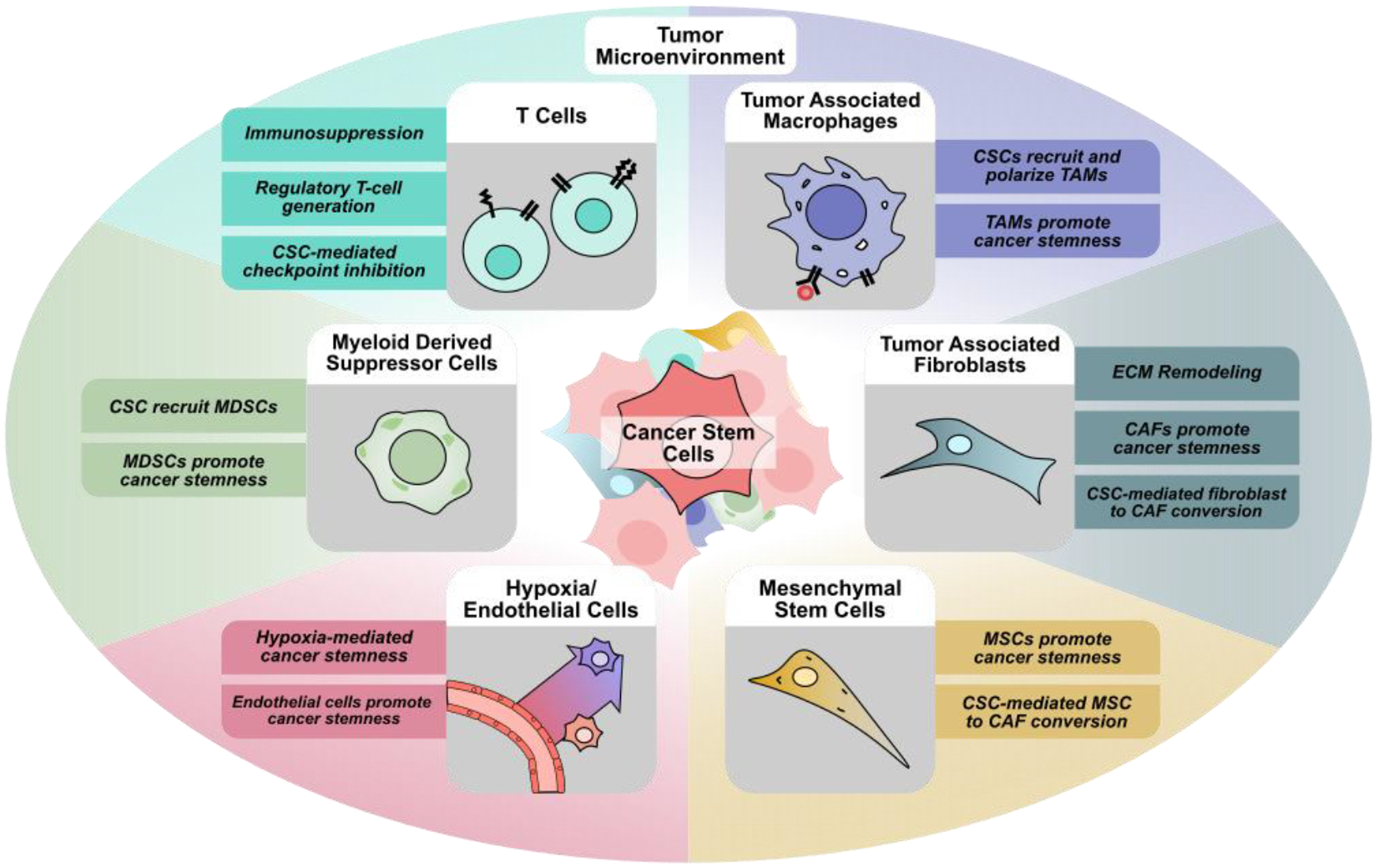
Interactions between cancer stem cells and other components of the tumor microenvironment. Within the tumor microenvironment, cancer stem cells interact with tumor associated macrophages (TAMs), cancer associated fibroblasts (CAFs), mesenchymal stem cells (MSCs), endothelial cells and areas of hypoxia, myeloid-derived suppressor cells (MDSCs), and other immune cells including CD4+ and CD8+ T-cells, regulatory T-cells, and natural killer cells. The ability for CSCs to actively recruit and interact with cells within the TME generates a pro-tumoral environment that supports cancer stemness, proliferation, metastasis, and immunosuppression.

Within tumors, cancer cells and CSCs do not exist in isolation, and are part of a complex network known as the tumor microenvironment (TME). The tumor microenvironment consists of a heterogeneous population of cells, including cancer, immune, and stromal cells, as well as other cells that make up larger structures within the TME such as blood vessels and nerves [12]. Initially thought to only play a passive role in tumor progression, these non-tumoral cells residing within the TME have been implicated in nearly all aspects of tumor biology (Figure 1). Numerous cell types such as fibroblasts, also known as cancer-associated fibroblasts, endothelial cells, mesenchymal stem cells, adipocytes, neurons, and immune cells drive tumor initiation, proliferation, metastasis, and cancer cell stemness [12]. Additionally, many immune cell types, such as CD8^+^ or CD4^+^ T cells, natural killer cells, dendritic cells, regulatory T cells, myeloid-derived suppressor cells, and macrophages have also been demonstrated to play an active role within the tumor microenvironment. Interactions between cancer cells and these immune cells also contribute to cancer stem cell maintenance, immune cell reprogramming, immune suppression, and are the basis for cancer immunotherapy [13]. Beyond its cellular components, the TME also encompasses the unique physiological conditions present within each tumor, such as hypoxia, low pH, and extracellular matrix stiffness [14]. Within the TME, the effects of different conditions combined with complex networks of signaling between the different cell types have been shown to regulate key features of cancer such as tumor proliferation, metastasis, and treatment resistance [15].

## 2. Cancer-Associated Fibroblasts and CSC Maintenance

**Figure 2:**
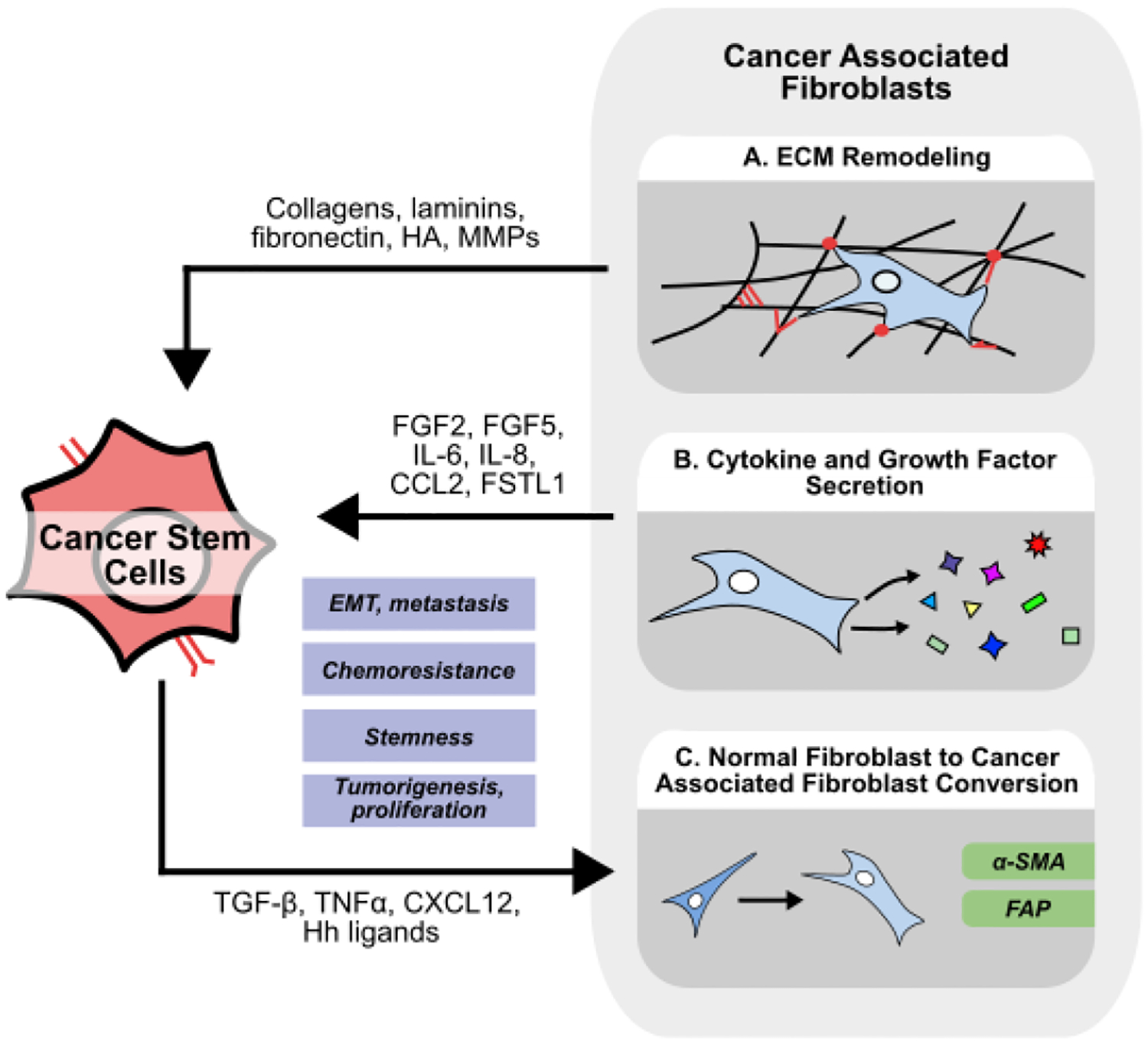
Crosstalk between cancer stem cells and cancer associated fibroblasts in the TME. (A) Cancer associated fibroblasts (CAFs) influence cancer cell stemness by modifying the extracellular matrix (ECM). CAFs produce structural proteins while also degrading and crosslinking the existing ECM. (B) CAFs secrete cytokines and growth factors to promote stemness in recipient cancer cells while also influencing proliferation, EMT & metastasis, and chemoresistance. (C) CSCs promote normal fibroblasts to take on a CAF-like phenotype marked by expression of α-SMA and FAP, while also influencing existing CAFs to produce pro-tumoral signaling molecules.

Found throughout the body, fibroblasts play a critical role in tissue repair, stroma homeostasis, and have been implicated in several diseases, including cancer [16]. To meet the diverse needs of the specialized tissues they reside in, fibroblasts display large inter-organ heterogeneity [17], but recent advances have also demonstrated a significant intra-organ and intra-tumor heterogeneity of fibroblasts [18,19]. Fibroblasts are well known for their ability to synthesize and maintain the extracellular matrix (ECM), and to secrete a variety of growth factors, cytokines, and chemokines to regulate other tissue-resident cells and processes (Figure 2) [16]. Within the tumor microenvironment, cancer-associated fibroblasts (CAFs) could be simply defined as fibroblast-like cells in close proximity to other cancer cells. However, in reality, distinguishing between normal fibroblasts and CAFs poses a challenge. Typically, CAFs are distinguished from normal fibroblasts by expression of alpha smooth muscle actin (α-SMA) and fibroblast activation protein (FAP), and display an altered secretome involving molecules such as IL-6 and CCL2 [20]. However, across different types of cancers, there are numerous suggested origins and mechanisms of activation for CAFs [21]. While many studies have shown that IL-1 and transforming growth factor-beta (TGF-β) are responsible for normal fibroblast to CAF transformation [22], CAFs may also originate from other cells such as mesenchymal stem cells.

### 2.1 CAF-mediated ECM remodeling

Within the tumor microenvironment, fibroblasts are the predominant regulator of the ECM, and are responsible for secreting, crosslinking, and remodeling structural proteins such as collagens, fibronectin, and laminins (Figure 2A) [23]. Apart from providing structure and stability within the TME, these structural molecules can bind many types of extracellular-sensing receptors on cancer cells to regulate their stemness [24]. For example, CAFs are known to secrete laminins such as laminin-332, which induces a stem-like phenotype in hepatocellular carcinoma (HCC), promoting quiescence and chemoresistance in an mTORC2-dependent manner [25,26]. Other laminins interact with integrins, such as integrin α6β4, on cancer cells to activate downstream Notch signaling, and promote cell proliferation and migration [27]. Similarly, activation of integrin ITGA2 on cancer cells upon interaction with collagen I promotes tumorigenesis and sphere formation in oral squamous cell carcinoma (OSCC) [28].

Other ECM components, such as fibronectin, interact with integrin αvβ3 on cancer cells to activate PI3K/Akt/SOX2 and CDC42/YAP-1/NUPR1/Nestin axes, promoting a stem-like phenotype in glioblastoma (GBM) [29]. Similarly, Wu *et al*. demonstrate that CAF-secreted fibronectin activates PI3K/Akt/SOX2 signaling in non-small cell lung cancer (NSCLC), and that inhibition of αvβ3 can sensitize cancer cells to chemotherapy [30]. Fibronectin also activates Wnt/β-catenin and MAPK/ERK signaling within NSCLC cells to promote angiogenesis and invasion in a WISP3-dependent manner [31]. Additionally, fibronectin-integrin interaction can upregulate the epithelial-mesenchymal transition (EMT) markers snail, N-cadherin, and vimentin [32], while fibronectin can promote cell migration, invasion, and matrix metalloproteinase 2 (MMP-2) expression in breast cancer [33]. CAF-derived MMPs are responsible for remodeling tumoral extracellular matrix, promoting angiogenesis, and degrading basement membrane to help promote EMT and invasion [34,35]. Given that MMP-7 has been shown to promote chemoresistance in numerous types of CSCs by mediating MUC-1 shedding [36], CAF-derived MMPs may also play a role in regulating stemness independent of canonical MMP-EMT pathways.

CAFs have been shown to secrete other structural molecules such as the polysaccharide hyaluronic acid (HA) in response to TGF-β [37]. In breast cancer, HA activates the stemness-related pathways Ras-MAPK and PI3K/Akt by binding to CD44, a widely acknowledged marker for CSCs [38,39]. Similarly, in head and neck squamous cell carcinoma (HNSCC), HA/CD44 mediated signaling enhances SOX2 expression through the PI3K/4EBP1 axis [40]. Apart from secreting structural proteins, CAFs can also regulate ECM stiffness by lysyl oxidase-mediated crosslinking of the existing ECM [41]. In HCC, matrix stiffening in 3D hydrogel culture is associated with increased expression of stemness markers CD44, EpCAM, and Nanog [42], while matrix stiffening promotes EMT in lung cancer in a DDR2-dependent manner [43]. Similarly, in colorectal cancer (CRC) cells, matrix stiffness is associated with increased CSC markers like CD133, ALDH1, and LGR-5, and knockdown of the Hippo transcription factor, Yes-associated protein (YAP), could reverse this effect and reduce sphere formation *in vitro* [44]. Together, these findings suggest that the ECM serves as more than a passive scaffold within the TME; the ECM is dynamically deposited and remodeled by CAFs to regulate cancer cell stemness via structural proteins and ECM-sensing receptors.

### 2.2 CAF-secreted growth factors

Apart from regulating the ECM, fibroblasts also secrete a variety of soluble factors to influence cancer cell migration, proliferation, and stemness (Figure 2B) [45]. Breast cancer-associated fibroblasts secrete fibroblast growth factor 5 (FGF5) to upregulate stemness markers ID3 and SOX10, and enhance sphere formation *in vitro* [46]. Similarly, breast cancer CAFs express high FGF2, and CAF-derived conditioned media promotes cancer cell proliferation *in vivo*, while knockdown of FGF2 in CAFs abrogated this effect [47]. Mechanistically, CAF FGF2 secretion is activated by estrogen via GPER/EGFR/ERK signaling [48]. Given that FGF2 helps maintain an undifferentiated state in mesenchymal stem cells marked by high expression of Oct4, SOX2, and Nanog [49], is also reported to convert iPSCs into CSCs, and is used in spheroid culture to enrich CSC populations [50,51], CAF-derived FGF2 may also play a larger role in regulating other cells within the TME.

Unsurprisingly, cancer cell and CAF communication is bidirectional. Cancer cells can secrete hedgehog ligands to promote FGF5 secretion from CAFs [46], while cancer cell-derived TGF-β can activate normal fibroblasts to adopt a more CAF-like phenotype (Figure 2C) [52]. In breast cancer, cancer cells secrete tumor necrosis factor α (TNFα) to induce CAFs secretion of hepatocyte growth factor (HGF), which promotes cancer cell proliferation, EMT, and radioresistance *in vitro*. Interestingly, radiation-exposed breast cancer cells secrete more TNFα to further enhance radioresistance in a CAF-dependent manner [53]. In gastric cancer, CAF-derived HGF promotes cell proliferation and migration in only Met-unamplified cells, while CAF-derived HGF enriched the proportion of CD44^+^ CRC stem cells *in vitro* and promoted metastasis through increased adhesion to endothelial cells *in vivo* [54,55]. In HCC, both fibroblast and cancer cell-derived TGF-β enhance expression of FSTL1 within CAFs. CAF-derived FSTL1 promotes tumorigenesis, metastasis, and sorafenib resistance *in vivo* [56]. In breast cancer, FSTL1 promotes stemness markers, sphere formation, and chemoresistance *in vitro*, while also promoting tumorigenesis and metastasis in gastric cancer [57,58]. In HCC, spatial multiomics identifies a subset of CAFs marked by high expression of FSTL1 that are localized within regions of cancer stemness. Unsurprisingly, these regions demonstrate enrichment of TGF-β and Notch signaling [59].

### 2.3 CAF-derived cytokines and chemokines

In gastric cancer, CAFs secrete IL-6 to promote EMT and metastasis by activating the JAK/STAT3 axis, while IL-6 promotes sphere formation and the expression of stemness markers Oct4, SOX2, CD44, and Nanog in HCC [60,61]. Similarly, breast cancer CAFs secrete IL-6 to promote radioresistance via STAT3 signaling, while CAF-derived IL-6 enhances p53 ubiquitination and degradation, allowing cells to escape doxorubicin-induced apoptosis [62,63]. High levels of other CAF-derived cytokines such as IL-8 are associated with poor response to chemotherapy and cisplatin resistance in gastric cancer [64]. In lung cancer, IL-8-secreting CAFs are marked by high levels of the filament Desmin, and are activated by cancer cell-derived lactate. Normal fibroblasts co-injected with lung cancer cells enhanced tumor growth *in vivo*, while knockout of the Desmin transcription factor JUNB-B in normal fibroblasts attenuated this effect [65]. Similarly, in ovarian cancer, CAF-secreted IL-8 promotes sphere formation and stemness *in vitro*, while CAF co-injection with ovarian cancer cells promotes tumor growth *in vivo* [66]. In OSCC, CAFs also produce the chemokine CXCL12 to increase sphere formation and expression of stemness markers in cancer cells [67]. CXCL12 has also been shown to stimulate normal fibroblasts to adopt CAF-like properties *in vitro* [68], suggesting that CAF secretion of CXCL12 may have a positive feedback effect. Given that CXCL12/CXCR4 signaling activates oncogenic ERK and PI3K/Akt signaling, and promotes chemotherapy resistance [69], it is unsurprising that high expression of CXCL2 is associated with metastasis to the brain, bone, lung, and liver in breast cancer [70]. Additionally, there are a number of other CAF-derived cytokines that regulate CSCs such as CAF-derived CCL2, which promotes CSC self-renewal via NOTCH1 signaling in breast cancer [71].

## 3. Immune Cells

**Figure 3:**
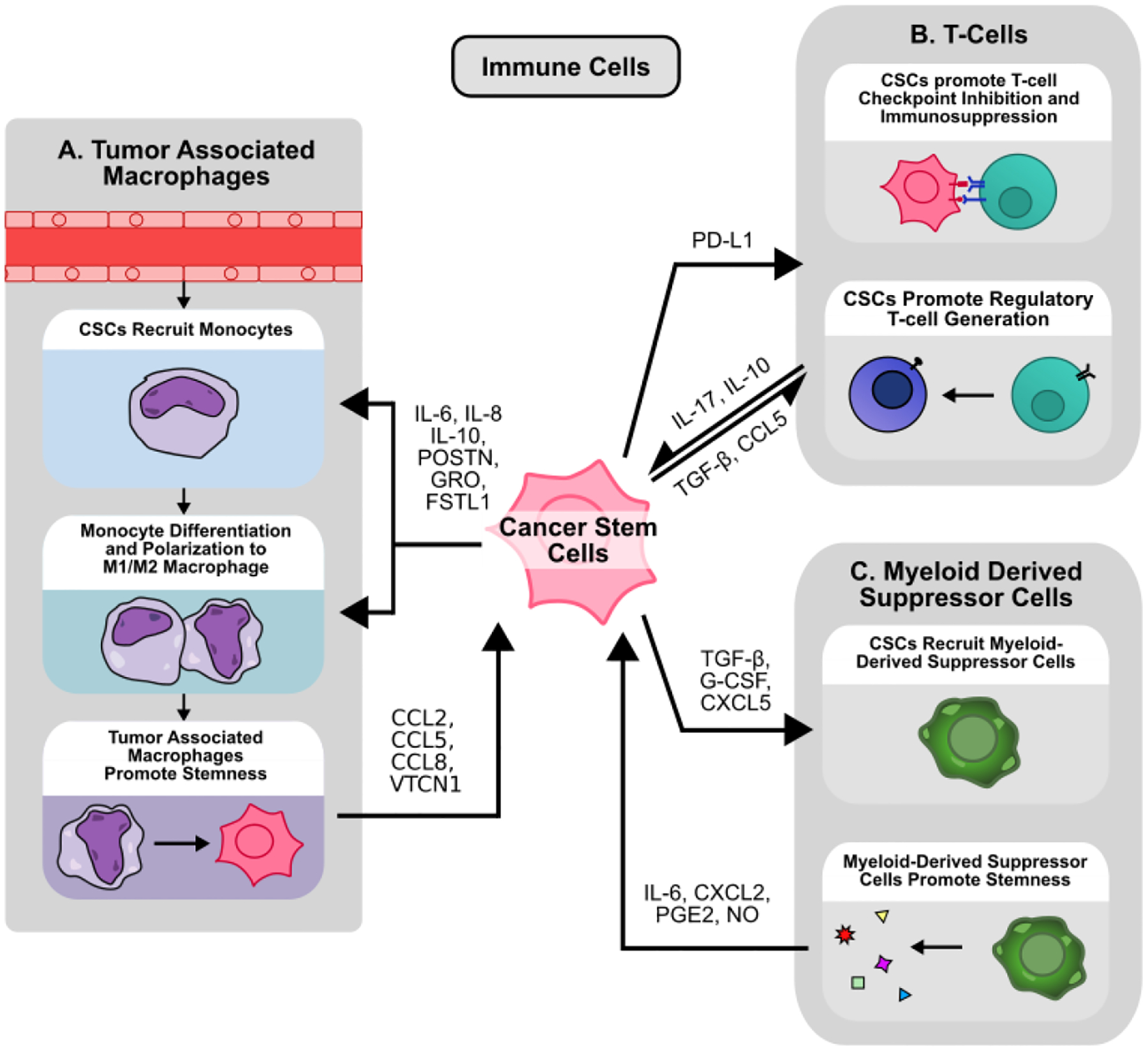
Crosstalk between cancer stem cells and immune cells in the TME. **(A)** CSCs mediate monocyte recruitment and polarization within the TME. CSC-derived cytokines such as IL-6, IL-8, and IL-10 control monocyte chemotaxis and polarization to an M1 or M2-like state. These tumor-associated macrophages (TAMs) can promote cancer cell stemness by secreting CCL2, CCL5, CCL8, and VTCN1. **(B)** CSCs promote immunosuppression via PD-L1 mediated checkpoint inhibition. CSC-derived TGF-β and CCL5 promote regulatory T-cell generation and recruitment. Resultant regulatory T-cells mediate immunosuppression via IL-10 while also promoting cancer stemness via IL-17. **(C)** Cancer stem cells (CSCs) actively recruit circulating myeloid derived suppressor cells (MDSCs) to the TME by secreting TGF-β, G-CSF, and CXCL5. These MDSCs can secrete IL-6, CXCL2, PGE2, and NO to promote cancer cell stemness.

Apart from CAFs, endothelial cells, and cancer cells, the tumor microenvironment also contains a heterogeneous population of immune cells [72]. At the most basic level, these immune cells can be classified either as tumor-promoting or tumorsuppressing [13]. CD8^+^ and CD4^+^ T cells are widely known for their ability to target and eliminate cancer cells [73], while other cells like natural killer (NK) cells, dendritic cells, and M1 macrophages also play a critical role in identifying and eliminating cancer cells within the TME [13]. On the other hand, regulatory T cells (Tregs), myeloid-derived suppressive cells (MDSCs), and M2 macrophages are recognized to be tumor-promoting, either by modulating other immune cells or interacting directly with cancer cells [13,74]. Unsurprisingly, within the TME, cancer cells and CSCs are not bystanders to immune cell activity. CSCs actively secrete signaling molecules to attract and modulate immune cell activity, and are likewise influenced by immune cell signaling. Moreover, CSCs also play an important role in escaping immune surveillance and inhibiting immune response in the TME (Figure 3) [75].

### 3.1 Tumor associated macrophages

Tumor associated macrophages (TAMs) are macrophages found within the TME, and originate either from tissue resident macrophages or circulating monocytes, the latter of which is the main source of TAM replenishment [76]. Monocyte-to-macrophage differentiation is driven by complex environmental cues and immune/cancer cell secreted cytokines such as colony stimulating factor (CSF) 1, CSF-2, and IL-34 [77]. Once differentiated, these monocytes can be further polarized by signals within the TME into two general categories, M1-polarized and M2-polarized macrophages. M1-polarized macrophages are recognized as tumor-suppressive, while M2-polarized macrophages are known for their tumor-promoting abilities [78]. However, these categories are likely an oversimplification, and macrophages *in vivo* exist on a spectrum of activation states influenced by a complex network of immune signals [79], of which CSCs play a critical role (Figure 3A). For example, in GBM, CSCs preferentially express periostin (POSTN) to recruit monocytes to the tumor site and promote polarization to an M2, tumor-promoting state via integrin αvβ3 signaling, a finding recapitulated in ICC [80,81]. Similarly, glioma stem cells (GSCs) induced STAT3 phosphorylation and STAT1 dephosphorylation in macrophages to promote M2-polarization and secretion of immunosuppressive IL-10 [82]. Inflammatory breast CSC-like cells also recruit monocytes and promote M2-polarization via IL-8 and GRO-mediated STAT3 phosphorylation in monocytes [83]. Additionally, numerous other studies have demonstrated the ability for CSCs to polarize monocytes to M2-like macrophages [84,85]. CD133^+^ GSC-derived IL-6 and IL-10 activate STAT3 signaling within macrophages to activate downstream expression of VTCN1. VTCN1 inhibits macrophage phagocytosis, T cell IL-2/IFN-γ production, and promotes T cell apoptosis, and silencing of VTCN1 in macrophages reduces tumor growth *in vivo* [86]. GSCs also preferentially express WISP1, which promotes stemness in an autocrine manner while promoting M2 TAM survival via integrin α6β1/Akt signaling [87]. Likewise, GSC-derived FSTL1 promotes stemness by activating PI3K-Akt signaling in an autocrine manner, while also promoting macrophage recruitment and M2 polarization *in vivo* [88].

CD14^+^ M2-like macrophages enhance sphere formation, stemness marker expression in GBM cells, and enrich the population of CD133^+^/CD44^+^ CSCs via CCL2 secretion. Co-injection of these M2-like macrophages with cancer cells enhances tumor growth *in vivo*, an effect reversed by inhibition of CCR2 [84]. Similarly, TAM-derived CCL8 promotes stemness of GBM cells cultured *in vitro*, while promoting tumor growth *in vivo*. Interestingly, cells cultured under sphere conditions overexpressed the CCL8 receptors CCR1 and CCR5 [89]. TAM-derived cytokines are also implicated in CSC regulation in numerous other cancer types. In breast cancer, high infiltration of CD163^+^ M2 macrophages in the TME is associated with lymph node metastasis and decreased survival, and TAM-derived CCL2 is responsible for enhancing the proportion of CD24^−^/CD44^+^ or ALDH^+^ CSCs via CCL2/Akt/β-catenin signaling *in vitro* [90]. Additionally, TAM-derived IL-6 promotes stemness via STAT3 signaling in HCC cells, and TAM-derived CCL5 similarly promotes stemness via β-catenin/STAT3 signaling in prostate cancer. Knockdown of CCL5 in TAMs inhibited tumor growth, metastasis, and CSC populations *in vivo* [91,92]. In HCC, spatial transcriptomic analysis suggests that CSCs are colocalized with SPP1^+^ macrophages within the TME, which are typically regarded as tumor-promoting M2-like macrophages [93,94]. Consistent with their role in tumor progression, SPP1^+^ macrophage and CSC colocalization is associated with poorer survival in patients with HCC. Interestingly, Fan *et al*. also show enhanced HIF-1α expression within colocalized areas [93]. Accordingly, HCC cells have been shown to secrete miR-1290 to induce M2-macrophage polarization [95]. Moreover, in HCC, SPP1^+^ macrophages are frequently found in close proximity to FAP^+^ CAFs [96,97], suggesting that macrophages, CAFs, and CSCs may form a complex network to regulate stemness within the TME. Given that M2 macrophages also suppress immune response in HCC by promoting the expression of exhaustion markers PD1/TIM3 on CD8^+^ T cells [98], CSCs may also modulate immune response via M2 macrophages.

While M1-macrophages, as the counterpart to M2-macrophages, were originally classified as tumor-suppressive, high levels of iNOS^+^ M1-polarized macrophages have been suggested to be associated with improved survival in HER2^+^ breast cancer [99]. However, Oshi *et al*. demonstrate that upon higher dimensional, transcriptomic classification of M1/M2 macrophages, M1 or the ratio of M1/M2 macrophages are not associated with improved survival in breast cancer [100], suggesting that the role of M1 macrophages may be more complex. *In vitro*, M1-polarized macrophages enrich the populations of CD24^−^/CD44^+^ and ALDH1^+^ breast CSCs during co-culture, while increasing the expression of stemness markers Lin-28B and Nanog in breast cancer cells [101]. Similarly, conditioned media from LPS/IFNγ-induced M1 macrophages enhances the expression of stemness markers in breast cancer cells via NFκB signaling [102]. In CRC, ID1-expressing M1-like macrophages have pro-tumorigenic and stemness promoting properties. Tumors grown in myeloid-specific ID1 knockout mice contain fewer ALDH^+^ and CD44^+^/Lgfr5^+^ CSCs, and inhibition of ID1 deubiquitination in M1 macrophages with ML323 reduces tumor growth, metastasis, and sensitizes tumors to chemo/immunotherapy [103]. These findings suggest that M1 macrophages, traditionally classified as tumor-suppressing, can also influence CSC maintenance and promote tumor growth.

### 3.2 CSCs and checkpoint inhibition

In addition to macrophages, CSCs also interact with other immune cells in the TME such as CD8^+^ and CD4^+^ T cells (Figure 3B). While activated CD8^+^ T cells are highly effective in clearing cancer cells, exhausted CD8^+^ T cells exhibit diminished effector function marked by high expression of checkpoint inhibitor receptors such as PD-1, CTLA-4, and TIM-3 [104]. While immunotherapies targeting these pathways can restore immune cell activity and enhance tumor clearance [105], CSCs have been shown to undermine these responses by mediating the expression of checkpoint inhibitors and activating additional mechanisms of immune evasion. For example, immunohistochemistry (IHC) analysis of CD44 expression, a common stem cell marker in lung adenocarcinoma (LUAD), reveals a positive association with PD-L1 expression [106], while CD133^+^/CD44^+^ CRC stem cells likewise exhibit higher PD-L1 expression via enhanced STAT3 signaling in patient-derived xenograft (PDX) models [107]. In breast cancer, CD24^−^/CD44^+^/EpCAM^+^ CSCs express higher levels of PD-L1 via Notch3/mTOR signaling *in vitro* [108], while ALDH1A1 expression by IHC in breast cancer is also associated with increased PD-L1 levels [109]. However, these associations may be context/cancer dependent. IHC analysis in NSCLC demonstrates a negative correlation between ALDH1 and PD-L1, and no significant relationship between CD44 and PD-L1 expression [110], suggesting that the relationship between stemness and PD-L1 expression may be more complex. Moreover, TGF-β-induced EMT increased PD-L1 levels in breast CSCs via β-catenin/STT3 glycosylation and stabilization of PD-L1 *in vitro* [111]. In HNSCC, IFN-I/IFNAR1 signaling promotes stemness and production of exosomal PD-L1 and Galectin-9, while IFNAR knockdown reduces the proportion of CD44^+^/ALDH^+^ CSCs, inhibits tumor growth, and promotes T-cell exhaustion *in vivo* [112]. However, CSC-derived PD-L1 not only acts on immune cells, but also on other cancer cells. In CRC, CD133^+^/CD44^+^ CSCs express higher levels of PD-L1, which interacts with HMGA1 to enhance sphere formation and Oct4 expression via Akt and MEK signaling [113], suggesting that CSCs can induce stemness within other cells in addition to suppressing immune response via PD-L1 expression.

### 3.3 Other immune cells

Apart from regulating CD8^+^ cells, CSCs also influence the activation and function of various other numerous immune cell populations. In breast cancer, CSCs promote NK cell migration but inhibit NK cell-mediated cytotoxicity. These CSCs express higher levels of NK-inhibiting ligand HLA-E, while downregulating the NK-activating ligands MICA and MICB [114]. However, other studies suggest that NK cells preferentially target CSCs in various cancers including breast cancer, glioma, sarcoma, and pancreatic cancer [115]. Additionally, CD4^+^ T cells cultured in the presence of breast CSC-conditioned media contain a larger proportion of CD4^+^CD25^+^CD127^−^ Tregs, possibly via CSC-derived TGF-β (Figure 3B) [116]. Breast CSCs also secrete exosomal FOXP3 to transform CD4^+^ T cells into CD4^+^/CD25^+^/FOXP3^+^ Tregs *in vitro* [117]. In GBM, CSCs not only promote the generation of Tregs, but also inhibit CD4^+^ and CD8^+^ cell activation and proliferation via activation of STAT3 signaling in T cells [118]. In HNSCC, CD44^+^ CSCs produce higher levels of numerous cytokines including TGF- β, and also promote Treg generation, MDSC generation, and inhibit T cell activation from PBMCs [119]. Ovarian CSCs not only induced Tregs to produce higher levels of the immunosuppressive cytokine IL-10, but also promoted the recruitment and chemotaxis of Tregs *in vitro* via CCL5 secretion [120]. *In vivo*, ovarian CD133^+^ CSCs express higher levels of the IL-17 receptor compared to non-CSCs, and colocalize with IL-17-producing cells such as CD4^+^ TH17 cells and macrophages. This IL-17 promotes sphere formation *in vitro* and tumorigenesis *in vivo* via NF-κB and p38 MAPK signaling in cancer cells [121]. Similarly, IL-17 promotes stemness in pancreatic cancer cells via NF-κB signaling [122].

### 3.4 Myeloid-derived suppressor cells

Other important immunosuppressive cells in the TME include MDSCs. MDSCs are a heterogeneous population of cells broadly classified for their immunosuppressive ability. MDSCs arise from myeloid progenitors in the setting of abnormal maturation and development, often due to cancer, chronic inflammation, or infection [123]. MDSCs are typically classified into two categories, polymorphonuclear and monocytic MDSCs (PMN-MDSC and M-MDSC). Although some typical surface marker sets are used to distinguish the two, higher dimension analysis has revealed many subpopulations within each category [124]. In the setting of cancer, MDSCs are recruited to the TME by chemokines such as CCL2 and CXCL12, where they can activate and proliferate via stimulation of STAT3, STAT6, and NF-κB pathways, among many others [125]. In breast cancer, CSCs preferentially express G-CSF via enhanced mTOR signaling, which results in MDSC accumulation within the TME [126], while melanoma CSC-derived TGF-β similarly recruits PMN-MDSCs to the tumor site [127]. In prostate cancer, Yap1-high CSC-like cells express higher levels of CXCL5, which promotes MDSC recruitment via CXCR2 signaling [128] (Figure 3C).

MDSCs are well-established mediators of T-cell immunosuppression, employing mechanisms such as L-arginine depletion, nitric oxide (NO) production, and reactive oxygen species (ROS) generation [129], but also regulate cancer cell stemness within the TME. In breast cancer, PMN-MDSCs enrich the proportion of ALDH^+^ CSCs, increase sphere formation, and upregulate expression of stemness markers via CXCL2/CXCR2 signaling [130]. Similarly, MDSCs cocultured with ovarian cancer cells enhance sphere formation ability and stemness markers via CSF-2/STAT3 signaling [131]. MDSC-derived IL-6 and NO activate STAT3 and NOTCH signaling pathways *in vitro* to enrich the population of ALDH^+^ CSCs in breast cancer [132]. Given that inducible nitric oxide synthase (iNOS) expression and subsequent NO production within cancer cells are associated with CSC maintenance via Notch1 signaling in numerous types of cancers [133,134], it is possible that MDSC-derived NO may exert a similar effect in cancers other than breast cancer. Similarly, while few studies have examined the role of MDSC-derived ROS in relation to CSCs, cancer cell-derived ROS are known to play multiple important roles in CSC maintenance, including ROS-mediated HIF stabilization [135]. In ovarian cancer, tumor-derived G-CSF promotes the generation and survival of MDSCs *in vivo* [136]. These MDSCs, in turn, secrete prostaglandin E2 (PGE2) to increase stemness and PD-L1 expression via the PI3K/Akt axis. Accordingly, G-CSF overexpressing tumors contain elevated levels of ALDH^+^ CSCs *in vivo* [136,137]. Additionally, in a mouse breast cancer model, PNM-MDSCs maintain the CD49f^+^ mouse CSC population via netrin-1 signaling. Notably, while anti-CTLA-4 immunotherapy enriched the population of CD49f^+^ CSCs, combination with netrin-1 blockade abolished this effect [138], suggesting that MDSC-mediated regulation of stemness may also contribute to therapy-induced CSC expansion.

## 4. Mesenchymal Stem Cells

**Figure 4:**
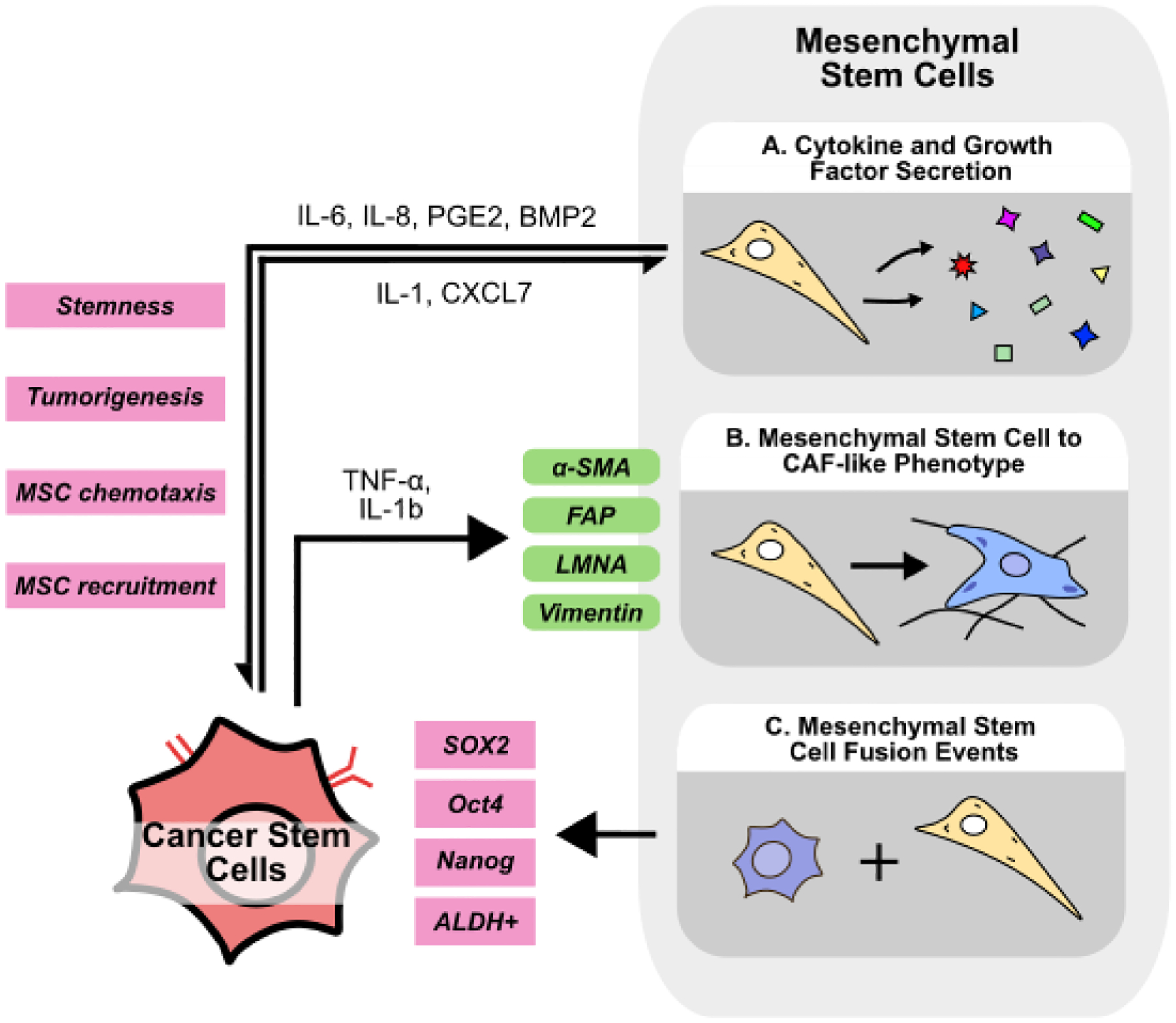
Crosstalk between cancer stem cells and mesenchymal stem cells in the TME. **(A)** Mesenchymal stem cells (MSCs) promote cancer cell stemness by secreting IL-6, IL-8, PGE2, and BMP2. MSC-mediated signaling is influenced by CSC-derived IL-1 and CXCL7. **(B)** CSCs can secrete TNF-α and IL-1 to influence MSCs to convert to tumor-promoting CAFs. **(C)** MSCs fused with normal cancer cells can become cancer stem cell-like cells marked by higher expression of SOX2, Oct4, Nanog, and ALDH.

Mesenchymal stem cells (MSCs) are multipotent stromal cells responsible for tissue repair, homeostasis, and immune response modulation. MSCs have been shown to differentiate into many cell types including adipocytes, osteocytes, and chondrocytes [139], and are present and able to be isolated from numerous types of tissue including bone marrow, adipose, placental, and umbilical cord tissue [140]. Isolated MSCs are typically characterized with markers such as CD105^+^/CD73^+^/CD90^+^ and CD45^−^, CD34^−^, MHC-II^−^, but MSCs are highly heterogenous and show variation both between and within tissue of origin [141]. Regardless, numerous clinical trials are investigating MSC isolation, expansion, and transplantation for a wide range of diseases [142]. MSCs secrete a variety of growth factors, cytokines, and miRNAs to promote tissue repair; however, these same molecules have also been implicated in cancer development and progression [143]. For example, MSC-derived TGF-β and PGE2 have been demonstrated to inhibit CD8^+^ T, NK, and dendritic cell-mediated inflammatory responses to cancer cells, and also to promote tumor-promoting M2-macrophage polarization [144]. Beyond immunosuppression, MSCs also play a pivotal role in mediating cancer cell stemness within the TME (Figure 4).

### 4.1 MSCs-secreted cytokines and chemokines

Numerous cytokines secreted by MSCs have been shown to promote stemness (Figure 4A). MSCs express higher levels of BMP2 upon coculture with ovarian cancer cells, which acts in a paracrine manner to increase the number of ALDH^+^/CD133^+^ CSCs and enhance sphere formation ability *in vitro*, an effect abolished upon treatment with the BMP signaling inhibitor, Noggin [145]. Furthermore, primary MSCs co-cultured with GSCs promote sphere formation *in vitro*, and tumorigenesis *in vivo* via MSC-derived IL-6/STAT3 signaling [146]. Similarly, MSCs isolated from human bone marrow (BM-MSCs) secrete IL-6 to promote cell invasion in HCC [147]. IL-6 has also been shown to enrich CSC populations and promote treatment resistance in several cancers including breast cancer, HNSCC, and colorectal cancer [148–150], suggesting that MSC-derived IL-6 may play a central role in promoting stemness across cancer types. In breast cancer, ALDH^+^ MSCs secrete CXCL7 to induce cancer cell IL-6 secretion, which can then promote MSC chemotaxis in a positive feedback loop. Accordingly, ALDH^+^ MSCs injected into the mouse tibia were targeted to the breast tumor site, promoting tumor growth and enriching the proportion of ALDH^+^ or CD24^−^/CD44^+^ CSCs *in vivo* [151]. Additionally, while MSCs are recognized to secrete PGE2 to promote recovery after liver injury [152], MSC-derived PGE2 also mediates CSC maintenance. When co-cultured with MSCs, CRC cells secrete IL-1, which stimulates MSCs to produce PGE2, IL-6, and IL-8. Co-injection of MSCs with CRC cells promotes tumorigenesis and enriches the ALDH^+^ CSC population via PGE2/Akt/β-catenin signaling [153]. PGE2 has also been demonstrated to promote cancer cell stemness in lung cancer via PI3K/Akt signaling, suggesting the effects of MSC-derived PGE2 may not be restricted to only CRC [154]. Similar to the findings reported by Liu *et al*. in breast cancer, BM-MSCs injected into the bloodstream are recruited into mouse prostate tumors, which then increase the number of CD44^+^/CD133^+^ CSCs. *In vitro*, co-culture of BM-MSCs with prostate cancer cells promotes sphere formation and the expression of stemness markers via downregulation of AR signaling [155]. In GBM mouse models, co-injection of MSCs with GBM cells likewise enhances tumor growth and enriches the population of tumoral CSCs [156]. In HCC, tumor-specific CD34^+^/CLDN5^+^ endothelial cell-derived IGF can recruit MSCs into the TME, which induces a stem-like phenotype in HCC cells [157], further supporting the notion that cells within the TME can actively recruit MSCs to enhance cancer cell stemness.

### 4.2 Other roles of MSCS

Apart from directly mediating cancer stemness, MSCs have also been suggested to convert to a CAF-like phenotype (Figure 4B). Long-term stimulation with TNF-α and IL-1b imparts MSCs with CAF-like morphology, increased vimentin and FAP expression, and an increased ability to contract collagen gels [158]. MSCs cultured in stiff, ECM-mimicking conditions acquire a CAF-like phenotype upon treatment with breast cancer cell-derived conditioned media. These CAF-like cells downregulate the MSC marker Stro-1 while upregulating CAF markers α-SMA and LMNA [159]. Similarly, transcriptomic analysis of MSCs treated with exosomes derived from colorectal cancer cells reveals an increase in CAF-related genes such as α-SMA and FAP [160]. Considering the substantial evidence supporting the role of CAFs in promoting stemness, context-dependent transformation of MSCs to CAFs may represent an additional mechanism of MSC-mediated enhancement of cancer stemness. Additionally, MSCs may also fuse with non-CSCs to generate CSC-like cells (Figure 4C). Dornin *et al*. provide evidence to suggest that breast cancer cells fused with MSCs contain a higher proportion of ALDH^+^ CSCs and also express higher EMT markers [161]. Similarly, lung cancer-MSC and normal epithelial cell GES1-MSC fusion hybrids express higher levels of EMT markers and possess increased tumorigenicity *in vivo* [162,163]. Fusion hybrids of GBM cells and MSCs demonstrate higher stemness markers SOX2, Oct4, and Nanog *in vitro*, while displaying increased tumorigenicity *in vivo* [164].

## 5. Exosomal Signaling and Stemness

**Figure 5:**
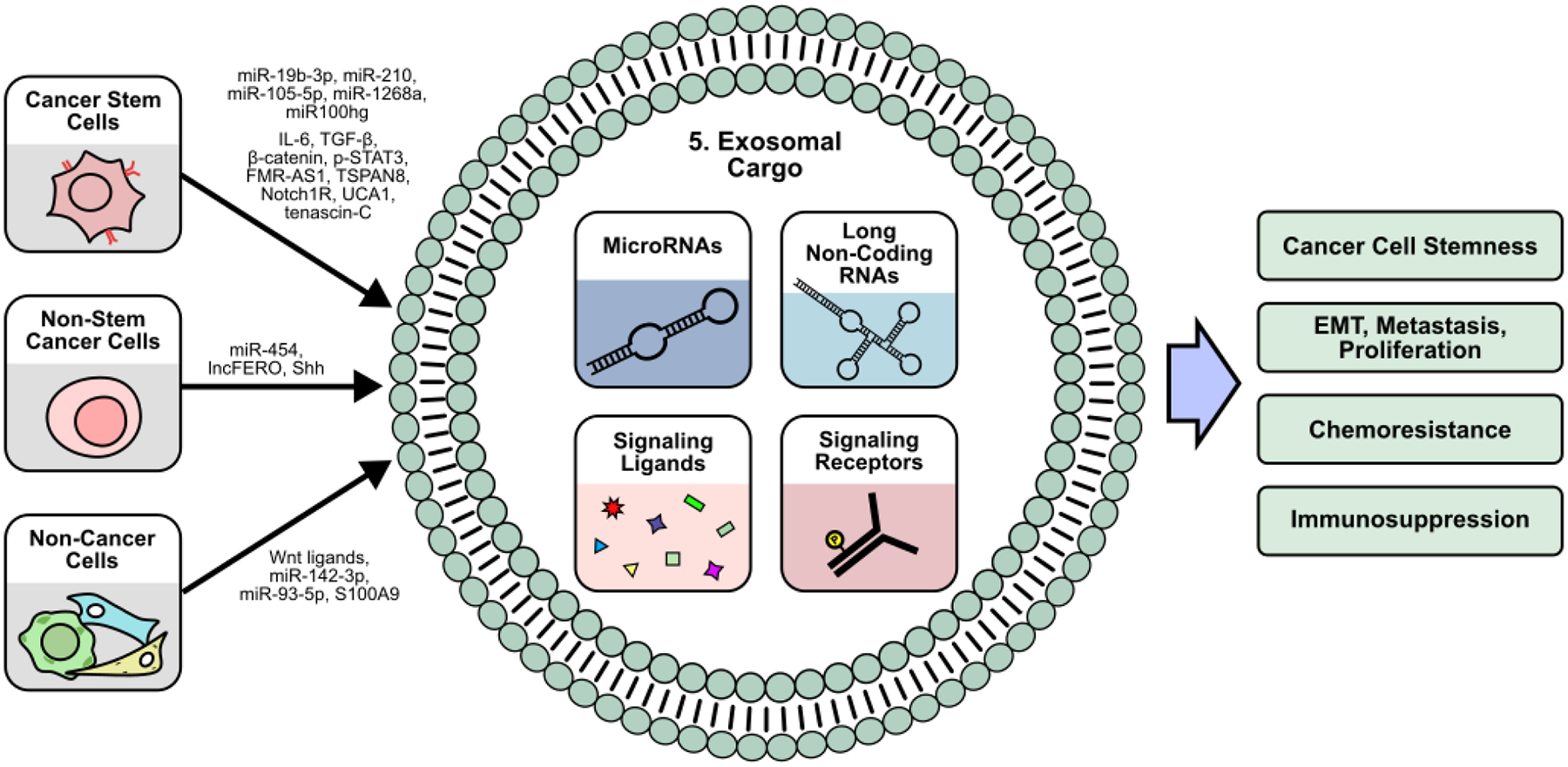
Cancer stem cell-related signaling within the TME is mediated by exosomes. Most cells within the TME, such as cancer stem cells, normal cancer cells, and non-cancer cells, including cancer-associated fibroblasts and mesenchymal stem cells, secrete exosomes to transport microRNAs, long non-coding RNAs, signaling molecules, and signaling receptors to other cells. Most cancer-relevant pathways such as those involving stemness, EMT, metastasis, proliferation, chemoresistance, and immunosuppression are regulated by exosomal cargo.

While a substantial portion of cellular signaling is mediated by small, soluble factors such as cytokines and chemokines, exosomes facilitate the transfer of larger macromolecules and insoluble factors, such as RNA, DNA, lipids, and proteins [165]. Exosomes are extracellular vesicles generated from the endocytosis of the plasma membrane, and thereby contain cell surface proteins that function to mediate their uptake and release [166]. Exosome formation is highly regulated and classified into ESCRT-dependent and independent pathways, which each consist of complex cascades governing cargo selection, membrane manipulation, and eventual exosome release [167]. As an important method of cell-to-cell communication, exosomes are critical to normal human development and cellular function, while exosomal dysregulation has been implicated in a wide variety of diseases including cancer [168]. Within the TME, exosomes and exosomal dysregulation have been shown to contribute to tumor proliferation, metastasis, and immune suppression (Figure 5) [169].

### 5.1 Cancer cell-derived exosomes

Additionally, exosomes have also been shown to promote stemness in recipient cells. For example, pancreatic ductal adenocarcinoma (PDAC) CSCs secrete exosomes enriched in tetraspanin 8 (TSPAN8) compared to their non-CSC counterparts. Exosomal TSPAN8 activates Hedgehog (Hh) signaling in recipient cells to promote colony formation, stemness marker expression, and tumorigenesis [170]. Similarly, in HCC, non-CSC-derived exosomes containing the Hh ligand Sonic hedgehog (Shh) could enhance recipient cell stemness [171]. In esophageal squamous cell carcinoma, CD44^+^ CSCs secrete exosomes containing the lncRNA FMR-AS1, which acts as a TLR7 ligand to promote TLR7/NF-κB mediated stemness in cancer cells [172]. In GBM, CSC-derived exosomes containing the Notch1 receptor enhance sphere formation, stemness marker expression, and tumorigenesis in recipient cells [173], suggesting that exosome-mediated stemness may not only be restricted to the transfer of ligands, but functional receptors as well.

In addition to exosomal proteins, exosomal RNA has also been widely studied in the context of CSCs. In breast cancer, exosomal miR-105–5p produced by CSCs promotes cancer cell migration and tumor growth by inhibiting the translation of GPR12 [174], while exosomal miR-454 derived from breast cancer non-CSCs activates Wnt signaling to expand the population of CD44^+^/CD133^+^ CSCs *in vitro* while enhancing tumor growth *in vivo* [175]. In melanoma, CSC-derived exosomes containing miR-1268a and miRNA-4535 have been shown to promote metastasis in non-CSCs by inhibiting autophagy [176,177], while lung CSCs secrete miR-210–3p via exosomes to promote EMT by targeting fibroblast growth factor receptor 1 (FGFRL1) translation in recipient cancer cells [178]. Similarly, in renal cell carcinoma (RCC), CSCs produce exosomal miR-19b-3p to promote EMT in recipient cells by inhibiting PTEN translation. Interestingly, these RCC CSC-derived exosomes have differential effects on tumor growth based on size and CD103^+^ incorporation, with high CD103^+^ exosomes being able to more efficiently target tumor cells [179]. miRNAs are known to be regulated by lncRNAs, which can sponge miRNAs to inhibit their activity [180], representing another layer of post-transcriptional regulation that CSCs can exploit to promote tumor progression. LUAD CSC-like cells produce exosomes enriched in the lncRNA Mir100hg, which can promote metastasis by enhancing ALDOA activity and N3K14 lactylation [181].

### 5.2 CSC exosome-mediated immune modulation

Recipients of CSC and non-CSC-derived exosomes are not only limited to cancer cells. CSC-derived exosomes have been demonstrated to mediate immune cell activity and promote immunosuppressive TME. While Wang *et al*. demonstrate that exosomal miR-210–3p can inhibit FGFRL1 in cancer cells [178], CSC-derived exosomes containing miR-210 also target macrophages in prostate cancer. Inhibition of FGFRL1 in macrophages promotes M2 polarization, while co-injection of these M2-polarized macrophages with prostate cancer cells promotes tumor growth and gemcitabine resistance *in vivo* [182]. In oral squamous cell carcinoma, CSCs produce exosomes containing the lncRNA UCA1, which inhibits miRNA-134-mediated silencing of laminin γ2 (LAMC2) in macrophages. The resulting upregulation of LAMC2 in macrophages and CD4^+^ T cells upon exosomal delivery of UCA1 promotes M2-polarization and reduces the proportion of activated IFNγ^+^/CD4^+^ T cells [183]. Similarly, tenascin-C, an ECM glycoprotein, is packaged into exosomes by glioblastoma stem cell-like cells to reduce T cell proliferation and activation via tenascin-C/α5β1 and αvβ6mediated signaling [184].

Furthermore, Gabrusiewicz *et al*. suggest that only monocytes and activated T cells are able to uptake glioblastoma stem cell-derived exosomes. Recipient monocytes exhibit M2-polarization and increased PD-L1 expression [185], suggesting that CSC-derived exosomes can selectively target certain immune cells within the TME. In CRC, CSC-derived exosomes localize to mouse bone marrow and induce bone marrow-derived neutrophils to secrete IL-1β. These IL-1β-secreting neutrophils exhibit increased lifespan and ability to enhance tumorigenicity of CRC cells *in vivo* [186]. Similarly, CRC CSCs produce exosomes enriched with β-catenin, IL-6, TGF-β1, and pSTAT3, which induce M2 macrophage polarization and normal fibroblast to CAF conversion, and reduction of this exosomal cargo upon ovatodiolide treatment reduces tumor growth *in vivo* [187]. *In vitro*, monocytes that differentiate into dendritic cells in the presence of RCC CSC-derived exosomes containing the human leukocyte antigen G (HLA-G) exhibit lower levels of dendritic cell activation markers CD83 and CD40, and the MHC-II molecule, HLA-DR [188].

### 5.3 CSC exosome-mediated chemoresistance

CSC-derived exosomes have also been suggested to mediate chemoresistance. Oral squamous cell carcinoma exosomes promote resistance to cisplatin *in vitro*, while reduction of exosomal cargo via ovatodiolide treatment sensitizes tumors to cisplatin *in vivo* [189]. Exosomes derived from gemcitabine-resistant pancreatic CSCs promote gemcitabine resistance in gemcitabine-sensitive cells by transferring miR-210 [190]. Similarly, in hepatocellular carcinoma, CSC-derived exosomes enhance regorafenib resistance *in vitro* in a Nanog-dependent manner [191], suggesting that CSC-derived exosomes may be another mechanism through which tumors can develop chemoresistance. Additionally, more substantial evidence demonstrates dysregulation of exosome production upon chemotherapy treatment. Breast CSC-like cells treated with cisplatin differentially express numerous miRNAs whose miRNA transcripts include numerous cancer-relevant proteins such as MYC and JAG1 [192]. Likewise, docetaxel and doxorubicin treatment of non-CSC breast cancer cells induces production of exosomal miRNAs targeting the transcription factor ONECUT2. While *in vivo* data suggests that ONECUT2 may promote tumor growth, miRNA-mediated downregulation of ONECUT2 upon docetaxel/doxorubicin treatment induces a stem-like phenotype by upregulating Notch1, Oct4, and SOX9 in breast cancer cells [193]. In gastric cancer cells, cisplatin and paclitaxel treatment induces exosomal lncFERO secretion, which promotes SCD1 mRNA translation to protect recipient CSCs against ferroptosis [194]. In bladder cancer, treatment of non-CSCs with gemcitabine/cisplatin promotes the production of exosomes that can protect recipient CSCs from chemotherapy [195].

### 5.4 Non-cancer cell-derived exosomes

Within the TME, the production of exosomes is not limited to cancer cells alone. In colorectal cancer, CAFs secrete exosomal lncRNA H19, which acts as a miR-141 sponge to promote tumor growth and resistance to oxaliplatin in recipient cells [196]. These non-cancer cell-derived exosomes have also been demonstrated to mediate stemness. In CRC, CAF-derived exosomes containing Wnt3a enrich stemness markers in cancer cells, promote their sphere formation ability, and drive in-vivo chemoresistance and tumor proliferation [197], while MDSC-derived exosomes containing S100A9 promote stemness of CRC cells *in vitro* and tumor growth *in vivo* [198]. Wang *et al*. demonstrate that S100A9 binds NADPH oxidase to increase ROS production and subsequently activate STAT3 and NF-κB signaling. Subsequent work by Wang *et al*. demonstrates that MDSC-derived exosomes also promote M2-macrophage polarization by transferring miR-93–5p to downregulate STAT3 signaling in recipient monocytes [199]. In addition to CAFs and MDSCs, BM-MSCs also regulate CSCs via exosomal signaling. BM-MSCs exosomes containing miR-142–3p promote sphere formation, stemness marker expression, and the proportion of CD133^+^/LGR5^+^ CSCs *in vitro*, while CRC cells overexpressing miR-142–3p exhibit increased tumorigenesis *in vivo*. Mechanistically, miR-142–3p targets mRNA transcripts of DUMB, a negative regulator of Notch signaling, to promote Notch pathway activity [200]. Similarly, in acute myeloid leukemia, BM-MSCs-derived exosomes upregulate S100A4 expression in recipient cancer cells, enhancing their sphere formation ability and expression of stemness markers [201]. However, BM-MSC exosomes containing the circular RNA circ-0030167 inhibit stemness *in vitro*, while also reducing tumor growth *in vivo* by inhibiting miR-338–5p/WIF1/Wnt8/β-catenin signaling in pancreatic cancer cells [202]. Altogether, these findings suggest that apart from CSCs and non-CSCs, various stromal and immune cells also contribute to the exosomal secretome within the TME.

## 6. Endothelial Cells

**Figure 6:**
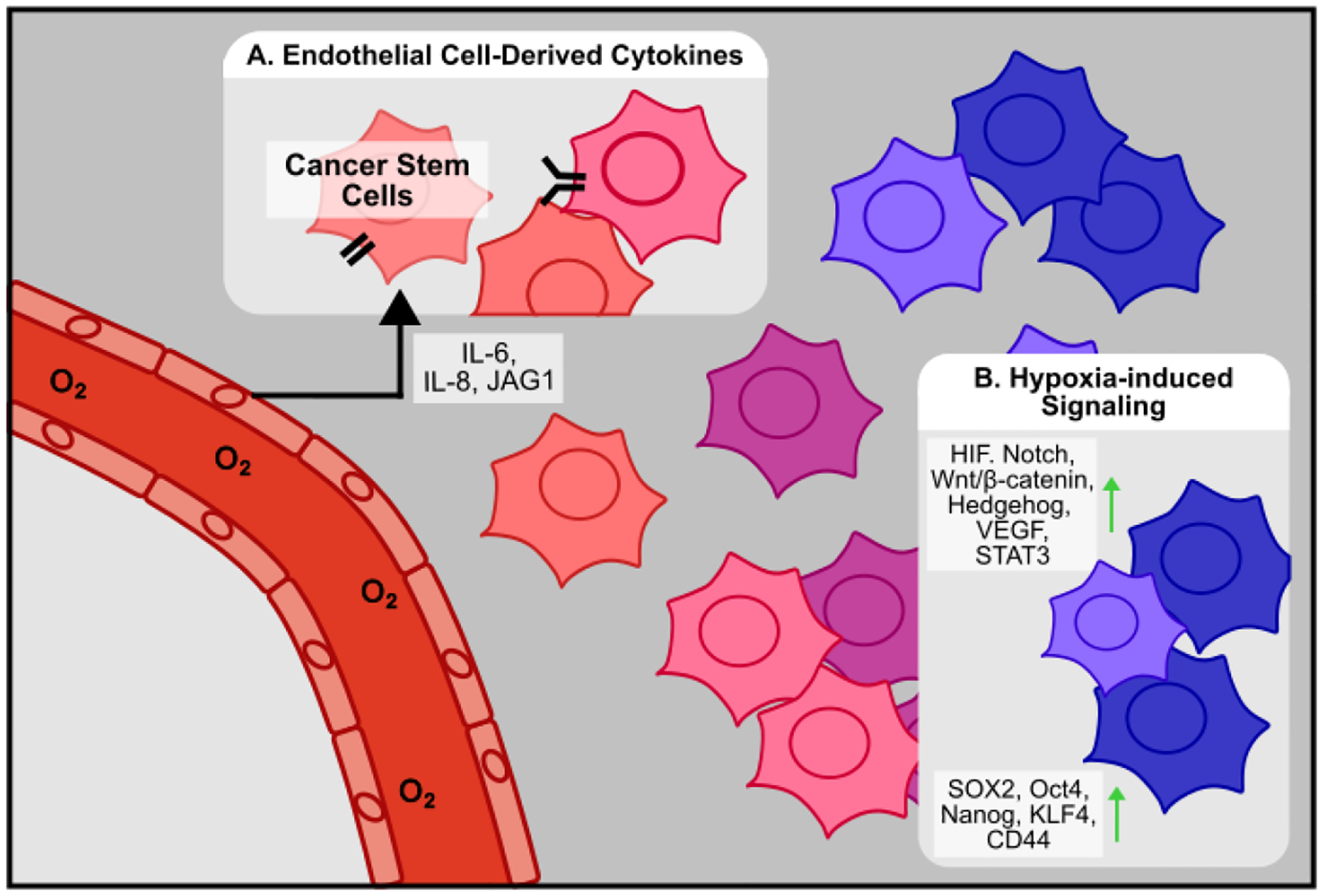
Cancer cell stemness is influenced by endothelial cells and hypoxia. **(A)** Endothelial cells secrete IL-6, IL-8, and JAG1 to promote cancer cell stemness. **(B)** In hypoxic regions, hypoxia-induced signaling by HIF and VEGF activates further downstream stemness-related pathways such as Notch, Wnt, Hh, and STAT3, ultimately resulting in higher expression of stemness markers such as SOX2, Oct4, Nanog, KLF4, and CD44.

Emerging evidence indicates that endothelial cells, beyond forming new vasculature, also actively contribute to CSC maintenance through direct and paracrine interactions (Figure 6A). In brain tumors, Nestin^+^/CD34^−^ CSCs are associated with endothelial cells *in vivo*, and also physically interact *in vitro*. Co-culture experiments demonstrated that endothelial cells enhance CSC self-renewal, while co-injection of CSCs and endothelial cells *in vivo* promotes tumor proliferation [203]. A similar phenomenon has been demonstrated in CRC, where endothelial cells are associated with CD133^+^ CSCs *in vivo*, enrich the proportion of ALDH1^+^ and CD133^+^ CSCs, and dedifferentiate cancer cells to CSCs via secretion of Notch ligand Jagged1 *in vitro* [204]. In GBM, endothelial cell-derived IL-8 is responsible for promoting stemness and tumor growth *in vivo* [205]. Similarly, in HNSCC, endothelial cells enhance cancer cell stemness via secretion of IL-6, and co-injection of endothelial cells with ALDH^+^/CD44^+^ CSCs increases tumorigenesis *in vivo* [206]. While ample evidence demonstrates the close association and interaction between endothelial cells and CSCs, Conigliaro *et al*. show that in HCC, CSC-derived exosomes containing the long noncoding RNA lncRNA H19 are responsible for promoting the attachment of CSCs to endothelial cells [207].

## 7. Hypoxia

Within the human body, oxygen is required for most biological processes. As such, cells have evolved mechanisms to sense and respond to low concentrations of oxygen, also known as hypoxia. The central hypoxia pathway involves the hypoxia inducible factor (HIF) family of transcription factors. The canonic HIF complex is composed of an HIF-α and an HIF-β subunit, which form a heterodimeric transcription factor to activate hypoxia-response translation [208]. In the most widely studied HIF complex, HIF-1, the β subunit HIF-1β is stably expressed while the α subunit HIF-1α exhibits oxygen-dependent regulation [209]. Under normal concentrations of oxygen, or normoxia, HIF-1α is hydroxylated at key residues, which allows a component of the E3 ubiquitin ligase, the von Hippel-Lindau (VHL) protein, to bind and target HIF-1α for proteasomal degradation. Under hypoxia, HIF-1α hydroxylation is inhibited, and the transcription factor is stabilized and free to complex with HIF-1β to regulate gene expression [210]. HIF regulates numerous cellular functions involving metabolism, gene transcription and translation, and angiogenesis [211]. However, given that hypoxia is a frequent characteristic of solid tumors, HIF signaling is, unsurprisingly, aberrantly activated within the tumor microenvironment to regulate many aspects of cancer biology, including cancer stem cell maintenance (Figure 6B) [212,213]. Within the tumor microenvironment, fast-growing cancer cells can outpace oxygen supply, leading to the development of hypoxic regions [214]. Given that hypoxia has been shown to be an important regulator of embryonic development by promoting dedifferentiation to a stem-like state [215], it is unsurprising that hypoxia has been shown to similarly regulate cancer stemness in the TME (Figure 6B). In glioblastoma, hepatocellular carcinoma, and lung cancer, hypoxia promotes expression of stemness markers *in vitro*. In sorted CD133^−^ non-CSC cells, long-term hypoxia treatment enriches the population of CD133^+^ CSC cells [216], suggesting that hypoxia may be a source of new stem-like cells within the TME. These findings have been reaffirmed in ovarian cancer, where hypoxia-induced NOTCH signaling enhances SOX2 expression, chemoresistance, and sphere formation [217]. Similarly, in GBM, hypoxia promotes the proliferation and maintenance of GSCs via an HIF-1α-dependent stabilization of the NOTCH ligand NICD [218]. Hypoxia has also been shown to regulate the stem-cell relevant Wnt/β-catenin pathway. In colorectal cancer, hypoxia activates Wnt/β-catenin signaling to promote sphere formation and the expression of stemness markers *in vitro* [219]. However, in GBM, hypoxia-dependent inhibition of the β-catenin/TCF-4 complex allows for the formation of the competing HIF-1α/TCF1/β-catenin complex to promote neuronal differentiation away from a stem-like state [220], suggesting that the numerous binding partners of HIF-1α and β-catenin complexes may act to fine-tune downstream gene expression in a tissue-dependent manner.

Hypoxia has also been shown to induce Hh signaling via HIF-1α [221]. In myeloid leukemia, CSCs rely on Hh signaling to maintain stemness [222]. Likewise, inhibition of Hh signaling in CD133^+^ GSCs decreases stemness markers and secondary sphere formation ability, suggesting that hypoxia-dependent Hh signaling may also play a role in GSC maintenance [223]. In pancreatic cancer, hypoxia can induce EMT through a mechanism that is Hh ligand independent, but dependent on HIF-1α and the Hh pathway protein Smoothened (SMO) [224]. Interestingly, hypoxia causes cancer cells to secrete the Hh ligand Sonic hedgehog (Shh), which acts on surrounding fibroblasts to synthesize fibronectin and collagen [225], suggesting that hypoxia may also play a role in mediating cancer cell-NF/CAF communication to further promote stemness within the TME. Apart from HIF-1α, HIF-2α has also been shown to contribute to CSC maintenance. In CRC, chronic hypoxia induces higher expression of HIF-2α, resulting in higher expression of stemness genes KLF4 and Oct4 [226]. In GBM, HIF-1α and HIF-2α both contribute to glioma cell dedifferentiation, while HIF-2α knockout decreases SOX2 expression and the proportion of CD133^+^/CD15^+^ CSCs [227]. Similarly, breast CSCs have also been shown to rely on HIF-2α for self-renewal and sphere formation ability, and HIF-2α knockdown *in vivo* synergizes with paclitaxel to suppress tumor growth [228]. While numerous stemness genes like SOX2 and Oct4 are likely regulated far downstream of HIF-1α, vascular endothelial growth factor (VEGF) contains a hypoxia response element (HRE) consensus sequence in its promoter and is known to be directly regulated by the HIF-1 transcription factor [229]. In triple negative breast cancer, VEGF promotes mammosphere formation, increases the proportion of ALDH^+^ CSCs, and upregulates SOX2 expression in a STAT3 and Myc-dependent manner [230]. Likewise, breast CSCs express higher levels of the VEGF receptor, Neuropilin-2 (NRP2) [231], further suggesting that the HIF-1α/VEGF/NRP2 axis contributes to stemness. Unsurprisingly, inhibition of NRP2 in breast CSCs inhibits self-renewal and mammosphere formation ability [232].

## 8. Conclusions

Within the context of the TME, CSCs and stemness appear to be highly dynamic. While CSCs may possess intrinsically higher activity in pathways that promote self-renewal and stemness, a multitude of signals from the TME also plays a significant role in maintaining stemness. Cytokines, growth factors, and other exosome-derived molecules such as miRNAs secreted by CAFs; MSCs; immune cells such as MDSCs, TAMs, and T cells; and other non-CSCs and CSCs contribute to the activation of numerous stemness-promoting pathways in CSCs. In turn, CSCs themselves contribute to the TME signaling landscape to recruit and polarize TAMs, convert normal fibroblasts to CAFs, promote angiogenesis, and suppress immune activity. Clearly, regulation of CSCs and stemness is a complex and reciprocal process.

While CSCs may originate during tumorigenesis from transformation of tissue-resident stem cells, dedifferentiation of epithelial or stromal cells, or other events such as cell fusion and EMT [233], the emergence of new CSCs and regions enriched in high-stemness cells may be driven by environmental cues from the TME. Similar to normal human stem cells, which exist in niches that support and regulate their ability to self-renew and differentiate, CSCs appear to depend on analogous supportive niches within the TME. For example, regulation of normal intestinal stem cells relies on the intestinal crypt, which provides a niche for stem cell development. Cells at the base of the intestinal crypt are exposed to high levels of Wnt, Notch, and EGF ligands, which contribute to the maintenance of stem-like properties [234]. Likewise, in the TME, regions of hypoxia may provide a niche for the development of stem-like cancer cells. Activation of HIF signaling and downstream Wnt/β-catenin signaling within cancer cells residing in these hypoxic regions generates cancer cells exhibiting stem-like properties. Within this hypoxic niche, these CSC-like cells may recruit other cells such as endothelial cells, which can further contribute to promoting stemness.

Like the diverse array of normal stem cell niches such as neural stem cells within the subventricular zone, hair follicle stem cells in the hair follicle bulge, and hematopoietic stem cells in the bone marrow, numerous environmental and cellular factors contribute to the varied mechanisms underlying CSC niches [234–236]. Notably, CSCs appear to possess the ability to actively create and remodel their own supportive niches within the TME. By recruiting circulating cells such as MDSCs, Tregs, and monocytes, and by reprogramming immune cells, converting normal fibroblasts to CAFs, and polarizing monocytes into tumor-promoting macrophages, CSCs can establish a microenvironment that reinforces their stem-like state. Within these constructed niches, these recruited/transformed cells are not only able to help maintain stemness, but also suppress immune activity, promote metastasis, contribute to treatment resistance, and overall contribute to tumor progression. Given this, targeting cancer stem cells appears to be a promising avenue for cancer therapy, and countless therapeutic strategies have been developed to directly target cancer stem cells. Two general approaches involve inhibition of CSC-related pathways such as Wnt, Notch, and Hh pathways, and immunotherapy-based strategies against CSC markers such as CD44, EpCAM, and CD33 [237,238]. While some have shown some promise in clinic, no CSC-based therapies have significantly transformed standard of care and outcomes [237]. As such, the crosstalk between CSCs and TME provides countless new targets for cancer therapy. Instead of directly targeting CSCs, targeting CAFs, TAMs, *etc*. within the TME can disrupt the niche that helps promote cancer stemness. Where directly targeting CSC-related pathways may be infeasible due to poor selectivity, undruggable targets, *etc*., targeting TME components that produce activators of such pathways can provide additional flexibility. Similarly, when CSC markers may be non-specific and easily lost, resulting in therapy resistance, targeting cellular markers within the TME could provide another more specific and stable target for reducing CSCs.

## Abbreviations

The following abbreviations are used in this manuscript:

ALDH: Aldehyde dehydrogenase
CAF: Cancer-associated fibroblast
CRC: Colorectal cancer
CSC: Cancer stem cell
CSF: Colony stimulating factor
ECM: Extracellular matrix
EMT: Epithelial-mesenchymal transition
FGF: Fibroblast growth factor
GBM: Glioblastoma
GSC: Glioma stem cell
HCC: Hepatocellular carcinoma
HGF: hepatocyte growth factor
Hh: Hedgehog
HIF: Hypoxia inducible factor
HNSCC: Head and neck squamous cell carcinoma
IHC: Immunohistochemistry
LUAD: Lung adenocarcinoma
MDSC: Myeloid-derived suppressive cell
M-MDSC: Monocytic MDSC
MMP: Matrix metalloproteinase
MSC: Mesenchymal stem cell
NK: Natural killer
NO: Nitric oxide
NSCLC: Non-small cell lung cancer
OSCC: Oral squamous cell carcinoma
PDAC: Pancreatic Ductal Adenocarcinoma
PGE2: Prostaglandin E2
PMN-MDSC: Polymorphonuclear MDSC
RCC: Renal cell carcinoma
Shh: Sonic hedgehog
TAM: Tumor associated macrophages
TME: Tumor microenvironment
TNFα: Tumor necrosis factor α
Treg: Regulatory T cell
